# The Army

**Published:** 1862-05

**Authors:** 


### THE ARMY.

Army Surgeons—The need of volunteers to the Surgical Staff of the Army to meet the emergencies which the destructive battles in different parts of the country now almost daily occasion, has led the Governors of many States to organize volunteer corps. In this State Gov. Morgan, with the aid of Surgeon-General Vanderpoel, has organized and commissioned an auxiliary corps, which is composed of the following gentlemen:—Drs. James R. Wood, Alfred C. Post, Ernest Krackowitzer, Stephen Smith, Charles D. Smith, George A. Peters, John O. Stone, Thaddeus M. Halstead, Willard Parker, Gurdon Buck, Lothar Voss, Thomas M. Markoe, and William Detmold, of New York City; Alden March, John Swinburne, and S. Oakley Vanderpoel, of Albany; Edward H. Parker, of Poughkeepsie; Charles Winne and S. B. Hunt, of Buffalo, and DeWitt C. Enos and Joseph C. Hutchinson, of Brooklyn.

---

We congratulate the medical staff of the Army, and the profession of the United States, on the passage of the Medical Reform Bill through Congress. Briefly, the Medical Bureau has gained these points:—1. A larger and more effective force; besides an addition to the force of the staff, it is now to have a special department of Sanitary Inspection, with a sufficient corps of officers to place our entire army under the constant sanitary surveillance of the Medical Bureau. 2. An increased rank, the Surgeon-General having now the rank of a Brigadier-General. 3. Finally, selection of the highest officers according to merit, and not the old effete system of succession by seniority, which was ever liable to place at its head a man incapacitated by age.

